# CD28 Deficiency Ameliorates Thoracic Blast Exposure-Induced Oxidative Stress and Apoptosis in the Brain through the PI3K/Nrf2/Keap1 Signaling Pathway

**DOI:** 10.1155/2019/8460290

**Published:** 2019-12-04

**Authors:** Peifang Cong, Changci Tong, Ying Liu, Lin Shi, Xiuyun Shi, Yan Zhao, Keshen Xiao, Hongxu Jin, Yunen Liu, Mingxiao Hou

**Affiliations:** ^1^College of Medicine and Biological Information Engineering, Northeastern University, No. 195, Chuangxin Road, Hunnan District, Shenyang l10016, China; ^2^Emergency Medicine Department of General Hospital of Northern theater command, Laboratory of Rescue Center of Severe Wound and Trauma PLA, No. 83, Wenhua Road, Shenhe District, Shenyang 110016, China; ^3^Institute of Metal Research, Chinese Academy of Sciences, No. 72, Wenhua Road, Shenhe District, Shenyang 110016, China

## Abstract

Blast exposure is a worldwide public health concern, but most related research has been focused on direct injury. Thoracic blast exposure-induced neurotrauma is a type of indirect injuries where research is lacking. As CD28 stimulates T cell activation and survival and contributes to inflammation initiation, it may play a role in thoracic blast exposure-induced neurotrauma. However, it has not been investigated. To explore the effects of CD28 on thoracic blast exposure-induced brain injury and its potential molecular mechanisms, a mouse model of thoracic blast exposure-induced brain injury was established. Fifty C57BL/6 wild-type (WT) and fifty CD28 knockout (CD28^−/−^) mice were randomly divided into five groups (one control group and four model groups), with ten mice (from each of the two models) for each group. Lung and brain tissue and serum samples were collected at 12 h, 24 h, 48 h, and 1 week after thoracic blast exposure. Histopathological changes were detected by hematoxylin-eosin staining. The expressions of inflammatory-related factors were detected by ELISA. Oxidative stress in the brain tissue was evaluated by determining the generation of reactive oxygen species (ROS) and the expressions of thioredoxin (TRX), malondialdehyde (MDA), SOD-1, and SOD-2. Apoptosis in the brain tissue was evaluated by TUNEL staining and the levels of Bax, Bcl-xL, Bad, Cytochrome C, and caspase-3. In addition, proteins of related pathways were also studied by western blotting and immunofluorescence. We found that CD28 deficiency significantly reduced thoracic blast exposure-induced histopathological changes and decreased the levels of inflammatory-related factors, including IL-1*β*, TNF-*α*, and S100*β*. In the brain tissue, CD28 deficiency also significantly attenuated thoracic blast exposure-induced generation of ROS and expressions of MDA, TRX, SOD-1, and SOD-2; lowered the number of apoptotic cells and the expression of Bax, cleaved caspase-3, Cytochrome C, and Bad; and maintained Bcl-xL expression. Additionally, CD28 deficiency significantly ameliorated thoracic blast exposure-induced increases of p-PI3K and Keap1 and the decrease of Nrf2 expression in the brain. Our results indicate that CD28 deficiency has a protective effect on thoracic blast exposure-induced brain injury that might be associated with the PI3K/Nrf2/Keap1 signaling pathway.

## 1. Introduction

Blast exposure is a public health concern all over the world, because it has caused massive injuries to military personnel and civilians in recent wars and conflicts [1]. Upon exposure to the blast wave of an explosion, extensive pulmonary, head, and internal injuries can occur that can lead to immediate death or other secondary severe injuries [2, 3]. The lung is susceptible to barotrauma caused by primary blast injury and is the focus of blast injury research [4–6]. Until recently, most studies have focused on injury to the region directly exposed to blast, while changes in other organs are often ignored. However, Cernak et al. found that blast waves can cause neuronal damage without direct injury to the head, and they hypothesized that blast waves could be transmitted into the brain through the major blood vessels of the chest, thereby leading to neurological effects that can be slow to appear [7]. Courtney et al. proposed two potential mechanisms for brain injury caused by a blast. One involves the kinetic energy of the blast wave transferred through the large blood vessels in the abdomen and chest to the central nervous system [8]. Importantly, vascular load induced by kinetic energy leads to both morphological and functional damage to distinct brain structures [9]. According to accumulating clinical and experimental evidence, Cernak et al. also found that systemic and local variations initiated by blast exposure significantly influence the brain's response and contribute to the pathobiology of acute or chronic deficits caused by the blast [9, 10]. Although cognitive and behavioral changes are caused by blast-induced neurotrauma, victims fail to exhibit the neuropathology expected to initially accompany a traumatic brain injury, which makes it difficult to detect the trauma [11].

CD28 (Cluster of Differentiation 28) is expressed in T cells and provides costimulatory signals required for T cell activation and survival [12]. T cell stimulation through CD28, in addition to the T cell receptor, can provide a potent signal for the production of various interleukins, such as IL-6. Mirzoeva et al. showed that a CD28 antagonist peptide significantly decreased the expression of IL-6 and COX-2 and the number of macrophages in irradiation-induced small intestine injury in mice [13], while Singh et al. showed that CD28 knockdown could decrease the expression of inflammatory factors, such as COX-2, IL-10, and IL-17A under UVB exposure [14]. In our previous study, CD28 deficiency caused a significant decrease in the severity of primary blast-induced renal injury by reducing the expression of IL-1*β*, IL-4, and IL-6 and increasing the level of IL-10 [15]. All these studies showed the CD28 is effective in initiating inflammation.

To demonstrate the effect of CD28 in brain injury after thoracic blast exposure, we studied oxidative stress and apoptosis changes in the brain at different times after blast injury. We also examined CD28 knockout (CD28^−/−^) mice to study whether CD28 could be a therapeutic target in thoracic blast exposure-induced brain injury.

## 2. Materials and Methods

### 2.1. Animals and Experimental Protocols

Fifty healthy C57BL/6 mice weighing 22 ± 2 g and aged 6-8 weeks were purchased from Beijing Vital River Laboratory Animal Technology Limited Company, P.R. China. Fifty healthy CD28^−/−^ C57BL/6 mice with same conditions were obtained from Jackson Laboratory (Sacramento, CA). All mice were kept in a room maintaining the temperature of 20 ± 2°C and humidity of 55–65% and allowed free access to food and water. Animal welfare and experimental design were approved by the Ethics Committee of the General Hospital of Northern Theater Command.

After acclimation, C57BL/6 mice and CD28^−/−^ C57BL/6 mice were separated and randomly divided into five groups with ten mice (from each of the two models) for each group: (1) control group, (2) 12 hours after blast exposure (12 h), (3) 24 hours after blast exposure (24 h), (4) 48 hours after blast exposure (48 h), and (5) 1 week after blast exposure (1 w). In addition to the control group, the other C57BL/6 and CD28^−/−^ mice were sacrificed at 12 h, 24 h, 48 h, and 1 w after the construction of the thoracic blast exposure model.

### 2.2. Establishment of Thoracic Blast Exposure Models

Mouse models of thoracic blast exposure-induced brain injury were established using a self-made explosive device as previously described [16]. In brief, it was designed by four parts: air compression device, fixture system, protection device, and data acquisition system. PCB pressure sensor (PCB, GE Company, USA) was used to record the instantaneous pressure and duration of the shock wave pressure (PSI) = voltage value∗1000/50.08. The instantaneous shock wave overpressure was 321 ± 24 PSI in this experiment.

### 2.3. Sample Collection

After 12 hours of fasting and 4 hours of water deprivation before the operation, mice were intraperitoneally anesthetized with 2% sodium pentobarbital (1.5 ml/kg). After serum collection, lung and brain tissues were donated. Half of each tissue was immersed in 10% formalin buffer for histological analysis, and the remaining part was placed in a nitrogen canister for protein determination.

### 2.4. Histopathological Assessment

Lung and brain tissues were excised, fixed in 10% formaldehyde buffer at room temperature, and embedded in paraffin by a Leica Microsystem tissue processor (ASP 300S, Germany). After sectioning at 3 *μ*m of thickness, they were stained with hematoxylin and eosin (HE) and examined under a light microscope.

### 2.5. Enzyme-Linked Immunosorbent Assay (ELISA)

Following the manufacturer's instructions, levels of TNF-*α*, IL-1*β*, and S100*β* in the mouse serum were detected by Enzyme-Linked Immunosorbent Assay (ELISA) kits (Nanjing Jiancheng Bioengineering Institute, Nanjing, People's Republic of China).

### 2.6. ROS Detection

Brain tissue was stained with 2,3-dimethoxy-1,4-naphthoquinone (1 : 100; cat. no. D5439; Sigma, USA) for fifteen minutes and then examined under a fluorescence microscope (Olympus, Japan).

### 2.7. TUNEL Detection

Terminal deoxynucleotidyl transferase dUTP nick end labeling (TUNEL) detection is widely used for apoptosis. Following the manufacturer's instructions, brain tissue was stained with TUNEL assay (Roche, Basel, Switzerland) for an hour and then examined under a fluorescence microscope (Olympus, Japan).

### 2.8. Western Blotting

Whole proteins were extracted from the brain tissues by the total protein extraction kit (Beijing Solarbio Science & Technology Limited Company, China), and the protein concentration was determined by the BCA protein quantitative kit (Hangzhou Fu De Biological Technology Limited Company, China). After adjusting to the same concentration, the protein samples were added to the corresponding proportion of SDS gel loading buffer, boiled and denatured for 5 min, underwent SDS-PAGE electrophoresis, transferred to 5% skim milk PBST buffer at room temperature for 1 h, and washed in PBST 3 times. Then, the appropriate primary antibody (Table 1) was added and incubated overnight at 4°C. The membrane was washed 3 times with PBST, and a horseradish peroxidase-labeled secondary antibody (Table 2) was incubated for 1.5 h at room temperature and then washed 3 times. Proteins were visualized using a Clarity Western enhanced chemiluminescence substrate (Bio-Rad Laboratories, Inc., Hercules, CA, USA) and a Tanon 5200 Full automatic chemiluminescence image analysis system (Tanon Science and Technology Co., Ltd., Shanghai, China). In the western blot images, one lane represented a pool obtained from several animals. But before this, we have done the western blot of a single animal, and the relative density was measured by that.

### 2.9. Immunofluorescence Staining

The brain was dewaxed with xylene, hydrated with a graded alcohol series, incubated with 3% H_2_O_2_ (80% methanol) for 30 minutes, and washed three times with PBS for 5 minutes each time. Antigen was repaired by a high pressure thermal antigen repair method. Samples were blocked with 10% goat serum for 30 minutes, incubated with primary antibody overnight at 4°C in a wet box, and stained with a fluorescent secondary antibody goat Anti-Rabbit IgG H&L Alexa Fluor® 488 (1 : 200; cat. no. ab150077; Abcam, Cambridge, UK) after washing three times with PBS for 5 minutes each time. Finally, samples were observed and photographed with a fluorescence microscope.

### 2.10. Statistical Analysis

All values are expressed as the means ± standard deviation (SD) and were analyzed using SPSS 20.0 statistical software (IBM, USA). All experiments were repeated at least 3 times. Significance was determined when *P* values < 0.05 were obtained by two-way ANOVA with Bonferroni posttest.

## 3. Results

### 3.1. Thoracic Blast Exposure Induced Inflammation and Increased the Expression of CD28 in the Lung

**Figure 1:**
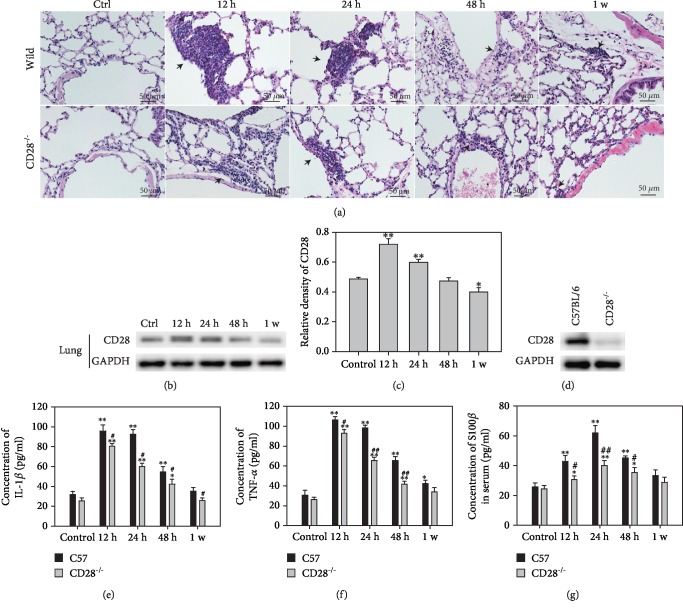
Inflammation and expression of CD28 increased in the lung after blast thoracic exposure. (a) Histopathological changes of the lung in C57BL/6 mice and CD28^−/−^ mice after blast thoracic exposure. The arrows point to the inflammatory region. (b) The expression of CD28 in the lung in C57BL/6 mice after blast thoracic exposure. (c) Relative density of CD28. (d) Western blot of CD28 in C57BL/6 mice and CD28^−/−^ mice. (e–g) The concentration of inflammatory factors detected in the serum by ELISA. Data are the mean ± SD. ^∗^*P* < 0.05 compared with the control group; ^∗∗^*P* < 0.01, compared with the control group; ^#^*P* < 0.05, compared with C57BL/6 mice in the same time point; ^##^*P* < 0.01, compared with C57BL/6 mice in the same time point.

After thoracic blast injury, inflammation was observed in the lung. As shown in Figure 1(a), histological staining of the lung clearly showed inflammation changes at 12 h and less inflammatory infiltration thereafter. CD28 was also detected as an important promoter of inflammation. Its level began to increase in the lung and peaked at 12 hours and then gradually decreased (Figures 1(b) and 1(c)). We engineered CD28 knockout mice and observed them at the same time points after thoracic blast exposure. The expression of CD28 in C57BL/6 and CD28^−/−^ mice was examined by western blot to ensure complete CD28 knockout (Figure 1(d)). In CD28^−/−^ mice, inflammatory cell infiltration was obviously diminished compared with C57BL/6 mice (Figure 1(a)). We also detected inflammatory factors in the serum.  Figure 1(e) shows that IL-1*β* increased rapidly with time and reached 95.69 ± 6.32 pg/ml in C57BL/6 mice at 12 h after thoracic blast exposure and then decreased gradually to 35.17 ± 3.53 pg/ml at 1 week. This increase was alleviated in CD28 knockout mice; IL-1*β* peaked at 80.6 ± 2.49 pg/ml at 12 h and then decreased successively at later time points, being significantly lower than the respective levels in C57BL/6 mice (*P* < 0.05). The concentration of TNF-*α* in the control group was 30.61 ± 4.9 pg/ml (Figure 1(f)). At 12 h after thoracic blast exposure, TNF-*α* level was 106.54 ± 3.34 pg/ml, which decreased to 42.29 ± 3.44 pg/ml at 1 week. The change in TNF-*α* levels followed the same trend in CD28^−/−^ mice as in C57BL/6 mice, but levels were significantly lower at 12 h, 24 h, and 48 h (*P* < 0.05). S100*β*, which is associated with brain injury [17–19], was also increased and peaked at 61.91 ± 4.97 pg/ml at 24 h in C57BL/6 mice after thoracic blast exposure, which was significantly higher than that in the control group (Figure 1(g), *P* < 0.05). In CD28^−/−^ mice, S100*β* levels increased to 40.03 ± 3.42 pg/ml at 24 h. These results demonstrate that inflammation can be induced by thoracic blast exposure and that this effect is ameliorated in CD28^−/−^ mice. The changes in serum S100*β* indicated the occurrence of brain injury.

### 3.2. Inflammation Is Diminished in the Brain of CD28^−/−^ Mice after Thoracic Blast Exposure

**Figure 2:**
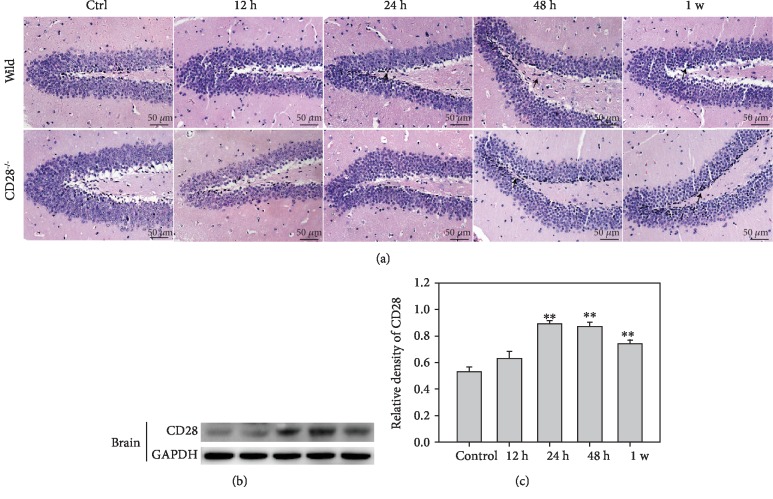
Inflammation and expression of CD28 increased in the brain after blast thoracic exposure. (a) Histopathological changes of the brain in C57BL/6 mice and CD28^−/−^ mice after blast thoracic injury. The arrow points to the inflammatory region. (b) The expression of CD28 in the brain in C57BL/6 mice after blast thoracic exposure. (c) Relative density of CD28. ^∗∗^*P* < 0.01, compared with the control group.

After thoracic blast exposure, inflammation in the brain was assessed by histological staining. In C57BL/6 mice, inflammatory cells presented most at 48 h, while less inflammatory cell infiltration was observed in CD28^−/−^ mice compared with C57BL/6 mice (Figure 2(a)). The level of CD28 protein in the C57BL/6 brain increased and peaked at 24 h (Figures 2(b) and 2(c)).

### 3.3. CD28 Deficiency Alleviates Oxidative Stress in the Brain Induced by Thoracic Blast Exposure

**Figure 3:**
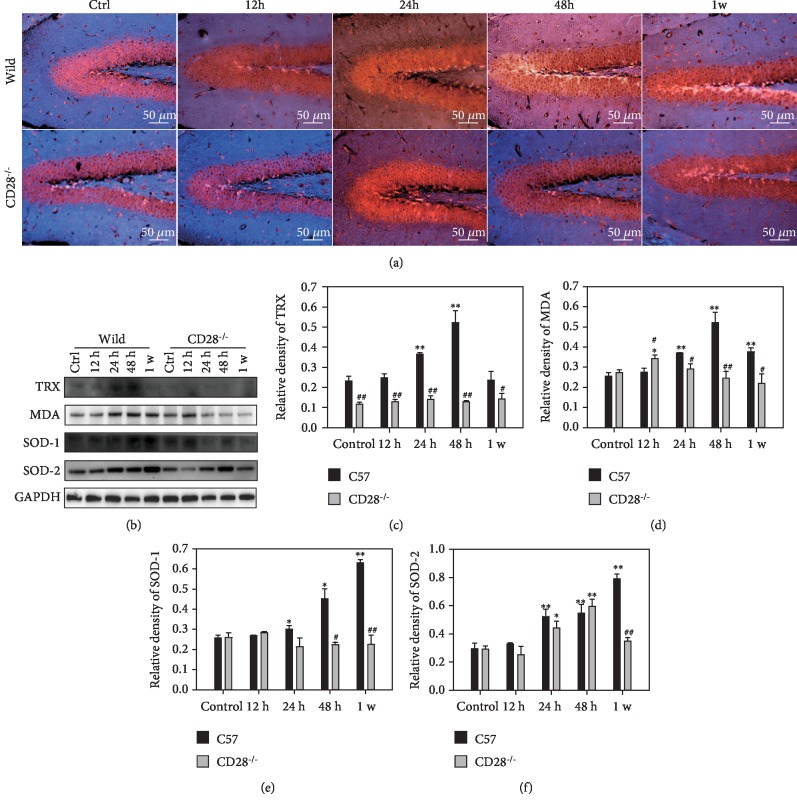
Oxidative stress in C57BL/6 mice and CD28^−/−^ mice with blast thoracic exposure. (a) ROS generation of brain tissue in C57BL/6 mice and CD28^−/−^ mice after blast thoracic exposure. (b) Western blot of intracerebral oxidant and antioxidant enzymes in C57BL/6 mice and CD28^−/−^ mice. (c) Relative density of TRX. (d) Relative density of MDA. (e) Relative density of SOD-1. (f) Relative density of SOD-2. ^∗^*P* < 0.05, compared with the control group; ^∗∗^*P* < 0.01, compared with the control group; ^#^*P* < 0.05, compared with C57BL/6 mice in the same time point; ^##^*P* < 0.01, compared with C57BL/6 mice in the same time point.

ROS generation indicates oxidative stress. Thoracic blast exposure caused ROS generation in the brain, which peaked at 24 h. CD28 knockout reduced ROS generation, as shown in Figure 3(a) by a decrease of red fluorescence and an increase of blue fluorescence.

Levels of intracerebral oxidant and antioxidant enzymes were examined by western blot. Thioredoxin (TRX) is a 12 kD oxidoreductase that responds to reactive oxygen species. Its level began to increase at 24 h and peaked at 48 h in C57BL/6 mice. In CD28^−/−^ mice, TRX was maintained at normal levels and was significantly lower than that of C57BL/6 mice at the same time points (*P* < 0.05, Figures 3(b) and 3(c)). As a final product of polyunsaturated fatty acid peroxidation, malondialdehyde (MDA) level is a marker for oxidative stress. The MDA level in the brain was elevated after thoracic blast exposure and reached a peak at 48 h in C57BL/6 mice. In CD28^−/−^ mice, the MDA level was increased at 12 h and then decreased, but the level was lower than that in C57BL/6 mice (*P* < 0.05, Figures 3(b) and 3(d)). Superoxide dismutase (SOD) is an important antioxidant in the event of oxidative stress. Western blotting demonstrated that SOD-1 and SOD-2 levels in C57BL/6 mice were increased after thoracic blast exposure but were slightly changed in CD28^−/−^ mice. In C57BL/6 mice, SOD-1 levels were increased at 12 h after blast thoracic exposure and then decreased with time. This increase was significant compared with the control group. SOD-2 levels increased till 48 h and then decreased. Both of these changes were significant compared with C57BL/6 mice at 1 w (*P* < 0.01, Figures 3(b), 3(e), and 3(f)).

### 3.4. CD28 Deficiency Alleviates Apoptosis in the Brain Induced by Thoracic Blast Exposure

**Figure 4:**
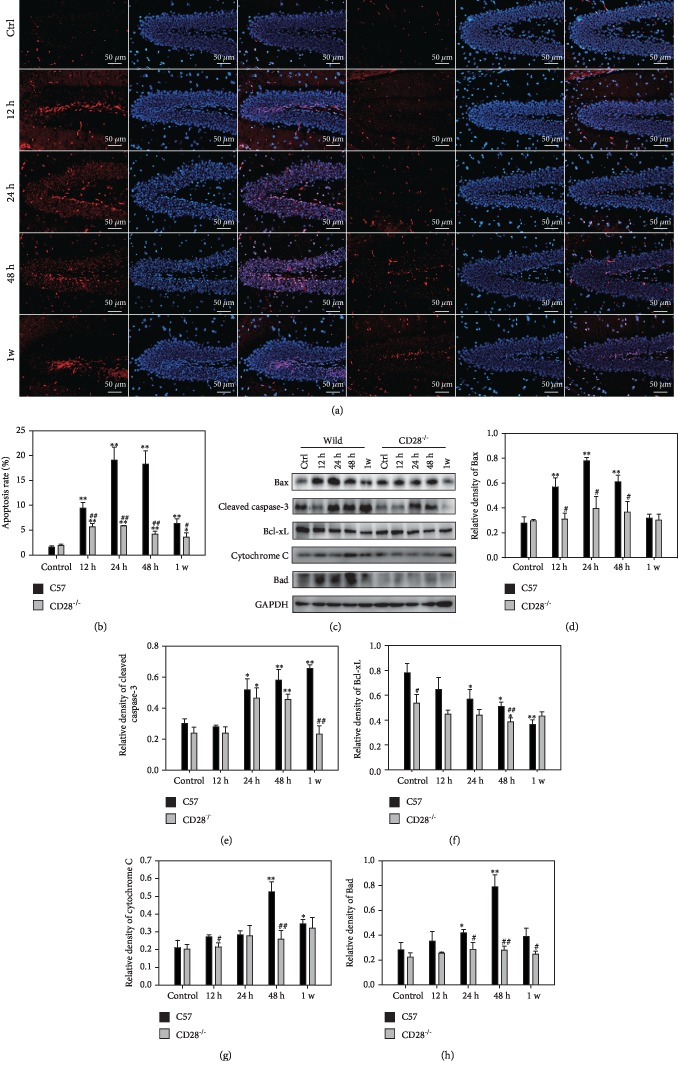
Apoptosis in C57BL/6 mice and CD28^−/−^ mice with blast thoracic exposure. (a) TUNEL of brain tissue in C57BL/6 mice and CD28^−/−^ mice after blast thoracic exposure. The cell with red fluorescence presented apoptosis. (b) Apoptosis rate of C57BL/6 mice and CD28^−/−^ mice. (c) Western blotting of proapoptosis and antiapoptosis proteins in C57BL/6 mice and CD28^−/−^ mice. (d) Relative density of Bax. (e) Relative density of cleaved caspase-3. (f) Relative density of Bcl-xL. (g) Relative density of Cytochrome C. (h) Relative density of Bad. ^∗^*P* < 0.05, compared with the control group; ^∗∗^*P* < 0.01, compared with the control group; ^#^*P* < 0.05, compared with C57BL/6 mice in the same time point; ^##^*P* < 0.01, compared with C57BL/6 mice in the same time point.

The TUNEL assay detects DNA fragmentation by labeling the 3′-hydroxyl termini of double-strand DNA breaks, which are generated during apoptosis [20]. The intensity of red fluorescence which presented apoptotic cell in the brain of blast model mice was higher than that in the C57BL/6 control group. And granulosa cells are TUNEL positive. The intensity of red fluorescence was lower in CD28^−/−^ mice at the same time points, which demonstrates that fewer cells were apoptotic and that the injury recovered more quickly (Figure 4(a)).

Western blot was used to examine the expression of proapoptosis and antiapoptosis proteins. Bax and Bad are proapoptotic members of the Bcl-2 family [21, 22]. Bax levels began to increase at 12 h and peaked at 24 h in C57BL/6 mice. In CD28^−/−^ mice, Bax levels peaked at 24 h but to a significantly lower level compared with C57BL/6 mice (*P* < 0.05, Figures 4(b) and 4(c)). Bad levels began to increase at 24 h and reached a peak at 48 h in C57BL/6 mice. But in CD28^−/−^ mice, Bad was maintained at its normal level and was significantly lower compared with levels in C57BL/6 mice (*P* < 0.05, Figures 4(b) and 4(g)). The caspase family plays a central role in the transduction of apoptotic signals. After thoracic blast exposure, the expression of cleaved caspase-3 in the C57BL/6 brain peaked at 1 w, but in CD28^−/−^ mice, it was increased at 24 h and then gradually decreased to a normal value (*P* < 0.05, Figures 4(b) and 4(d)). Levels of Bcl-xL, a member of the antiapoptotic Bcl-2 family, decreased with time in the brain of C57BL/6 mice after thoracic blast exposure. However, in CD28^−/−^ mice, Bcl-xL levels were maintained at the 12 h level (*P* < 0.05, Figures 4(b) and 4(e)). Cytochrome C is released from mitochondria before the morphological changes associated with apoptosis [23]. Cytochrome C levels increased and reached a peak in C57BL/6 mice at 48 h after thoracic blast exposure. In CD28^−/−^ mice, Cytochrome C levels increased but to a much lower level compared with C57BL/6 mice (*P* < 0.01, Figures 4(b) and 4(f)).

### 3.5. CD28 Deficiency Inhibits PI3K and Activates Nrf2/Keap1 Signaling to Alleviate Brain Injury following Thoracic Blast Exposure

**Figure 5:**
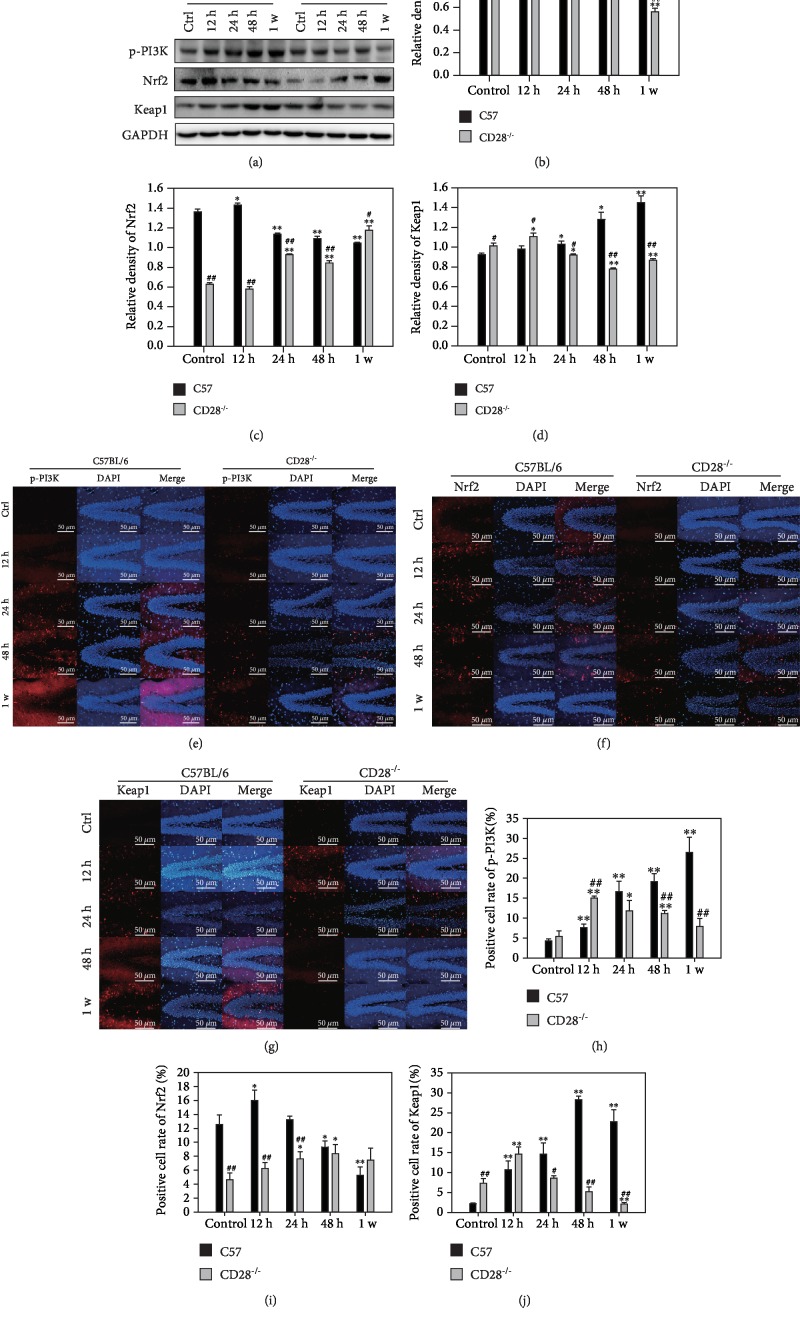
Expression of the PI3K and Nrf2/Keap1 signaling pathway in C57BL/6 mice and CD28^−/−^ mice with blast thoracic exposure. (a) Western blot of the PI3K and Nrf2/Keap1 signaling pathway in C57BL/6 mice and CD28^−/−^ mice. (b) Relative density of p-PI3K. (c) Relative density of Nrf2. (d) Relative density of Keap1. (e) Immunofluorescence of p-PI3K. (f) Immunofluorescence of Nrf2. (g) Immunofluorescence of Keap1. (h) Positive cell rate of p-PI3K in immunofluorescence. (i) Positive cell rate of Nrf2 in immunofluorescence. (j) Positive cell rate of Keap1 in immunofluorescence. ^∗^*P* < 0.05, compared with the control group; ^∗∗^*P* < 0.01, compared with the control group; ^#^*P* < 0.05, compared with C57BL/6 mice in the same time point; ^##^*P* < 0.01, compared with C57BL/6 mice in the same time point.

To examine oxidative stress and apoptosis-related signaling, we analyzed PI3K and the Nrf2/Keap1 pathway by western blotting. The expression of p-PI3K increased and reached a peak at 48 h in C57BL/6 mice. However, in CD28^−/−^ mice, the level of p-PI3K tended to decrease, which was significantly different from that in C57BL/6 mice (*P* < 0.05, Figures 5(a) and 5(b)). Nrf2 tended to gradually decrease with time in C57BL/6 mice, but tended to increase in CD28^−/−^ mice (*P* < 0.05, Figure 5(c)). The expression of Keap1 increased with time in C57BL/6 mice, while in CD28^−/−^ mice, it was increased at 12 h and then decreased after 24 h (*P* < 0.05, Figure 5(d)). Immunofluorescence and western blotting results for p-PI3K, Nrf2, and Keap1 were consistent (Figures 5(e)–5(g)).

## 4. Discussion

The major findings of this study are that thoracic blast exposure induced the following: (i) brain injury, caused by increased inflammation in the brain and increased levels of serum inflammatory factors; (ii) an oxidative stress response, as indicated by ROS generation and the expression of intracerebral oxidant and antioxidant enzymes; (iii) apoptosis, as indicated by TUNEL assays and the expression of proapoptosis and antiapoptosis proteins; (iv) activation of PI3K/Nrf2-Keap1 signaling; and (v) all the above changes which were alleviated by CD28 deficiency. These findings indicate that thoracic blast exposure induces oxidative stress and apoptosis in the brain via CD28 and the PI3K/Nrf2/Keap1 signaling pathway.

Traumatic brain injury-induced acute lung injury has been reported in many studies. Although the specific pathogenesis for this remains unclear [24, 25], some studies indicate that inflammatory factors can lead to injuries in undamaged organs by acute effects on leukocyte chemotaxis and vascular barrier disruption [26–28]. As the inflammatory response is the main pathological change during the development of acute lung injury [29–31], it may also be responsible for brain injury after by thoracic blast exposure. Some studies suggest that blast-induced neurotrauma can be induced by coeffects of the blast exposure and systemic and local responses and that multiple mechanisms are involved [32–34]. Here, we observed inflammation in lung tissue and increased levels of serum inflammatory factors, TNF-*α*, IL-1*β*, and S100*β*, after thoracic blast exposure. Inflammation in the hippocampal region of the brain at later time points also indicated the occurrence of brain injury. The occurrence of inflammation in the unexposed organs demonstrated that brain tissue can be impaired by thoracic blast exposure.

CD28 is one of the most important costimulatory signals that can promote the activation and survival of T cells. It can also lead to the production of various interleukins as a potent signal for stimulating T cells [35, 36]. By playing a very important role in the initiation, maintenance, and downregulation of the immune response, CD28 signaling is a key factor in many diseases [37–39]. Laurent et al. found that blockade of CD28 signaling prevented the development of lupus nephritis and prolonged survival [40]. We, therefore, predict that CD28 deficiency may ameliorate thoracic blast exposure-induced brain inflammation. Our results of the CD28 knockout group were consistent with this prediction. Decreased inflammation was observed in the hippocampus of the CD28^−/−^ group at a later time point compared with that of the C57BL/6 group (Figure 2). The reduced generation of ROS and decreased apoptosis at different time points also demonstrated that CD28 deficiency can reduce brain injury and lead to quicker recovery.

As a family of intracellular signal transducing enzymes, PI3Ks are involved in many cellular functions, such as cell proliferation, differentiation, survival, and intracellular trafficking. Gomez et al. found that PI3K-mediated signaling was stimulated in the protection of astrocytes from ceramide-induced apoptosis [41]. In our results, the phosphorylation (activation) of PI3K was increased (Figure 5). As an upstream signal, CD28 possesses an intracellular domain containing several residues that are critical for its signaling function, for example, recruitment of SH2 domain-containing proteins, especially PI3K [42], Grb2 [43], and Gads [44]. In our study, CD28 deficiency decreased the phosphorylation of PI3K (Figure 5).

Nuclear factor erythroid 2-related factor 2 (Nrf2), a bZIP transcription factor, plays a well-established role by regulating a battery of antioxidant and cell stress genes against oxidative stress [45]. It also plays an important role in immune responses and T cell activation in numerous rodent and primary human cells. Under normal conditions, Nrf2 is maintained at low levels by rapid ubiquitylation and proteasome-dependent degradation. However, during oxidative stress, this degradation can be inhibited in multiple organs, such as the liver, heart, kidney, and brain [46–53]. This depends on Keap1, which colocalizes with Nrf2 in the cytoplasm, and oxidation of critical Keap1 cysteine residues [54]. This allows Nrf2 to bind to the promoters of several antioxidant genes to protect against injuries by Keap1-Nrf2 dissociation and activation of Nrf2 [55, 56]. Sun et al. found that melatonin upregulates Nrf2 to protect against early brain injury after subarachnoid hemorrhage [57]. Xu et al. found that Nrf2 activation in astrocytes contributes to spinal cord ischemic tolerance induced by hyperbaric oxygen preconditioning [58]. Miller et al. showed that nrf2 antioxidant response elements mediated gene targets in the cortex and hippocampus after controlled cortical impact traumatic brain injury in mice [59]. Many studies have also confirmed that regulating the PI3K/Nrf2 pathway can alleviate acute renal injury and acute lung injury in the rat, oxidative damage in gastric epithelial cells, and cerebral ischemia-reperfusion-induced neuroinflammation and oxidative stress [60–62]. Li et al. found that paeonol and danshensu combination attenuated apoptosis in myocardial infarcted rats by inhibiting oxidative stress through the Nrf2/HO-1 and PI3K/Akt pathway [63]. Our findings show that Nrf2 was initially upregulated and then gradually downregulated and that Keap1 was upregulated in the brain via PI3K phosphorylation after thoracic blast exposure. However, in CD28^−/−^ mice, Nrf2 increased with time, which was different from Keap1 (Figure 5).

Oxidative stress is involved in many injuries. Many intracellular signaling pathways can be influenced by the excessive accumulation of ROS, which promotes the dissociation of Nrf2 and Keap1. This dissociation is mainly dependent on Keap1 activity or Nrf2 phosphorylation, which is associated with the activation of the kinases, for instance PI3Ks [64–68]. Subsequently, oxidant and antioxidant enzymes, such as CAT, TRX, SOD, and NADPH dehydrogenase quinone 1 (NOQ1), are further activated [69, 70]. Our findings show that ROS accumulate in the brain after thoracic blast exposure. The levels of intracerebral oxidant and antioxidant enzymes also demonstrated oxidative stress in the brain and that CD28 knockout could attenuate these changes (Figure 3).

Oxidative stress related to Nrf2 is often accompanied by apoptosis. In chronic obstructive pulmonary disease patients, the level of Nrf2 in lung tissue is reduced, and the decline in NRF2-dependent proteasomal activity was the reason for increasing apoptosis [71–73]. Sun and colleagues found increased Nrf2 expression after subarachnoid hemorrhage. Levels of the apoptosis-related proteins, Bax and cleaved caspase-3, were increased while those of the antiapoptosis protein, Bcl-2, were decreased [57]. In our study, apoptosis was observed in the brain by TUNEL staining. The levels of proapoptotic proteins, Bax, Bad, and cleaved caspase-3, were elevated, and those of the antiapoptotic protein, Bcl-XL, were reduced. All these apoptotic changes were alleviated in CD28^−/−^ mice (Figure 4).

**Figure 6:**
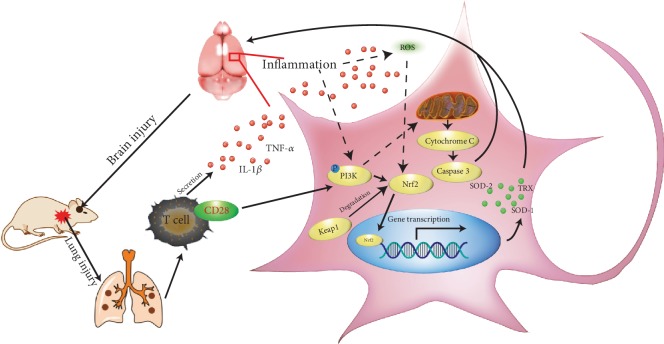
CD28 regulates blast thoracic exposure-induced oxidative stress and apoptosis in brain tissue in mice via the PI3K/Nrf2/Keap1 signal pathway.

From the above findings, we infer that thoracic blast exposure can lead to a systemic inflammatory response via upregulation of CD28, resulting in brain injury. ROS generation and the phosphorylation of PI3K were observed, and Nrf2/Keap1 signaling was activated. Although the levels of antioxidant enzymes were increased, higher levels of oxidative stress and PI3K phosphorylation still led to increased expression of proapoptosis and antiapoptosis proteins and apoptosis (Figure 6). The alleviation of these changes in CD28^−/−^ mice demonstrated that CD28 is a key factor in brain injury caused by thoracic blast exposure.

## 5. Conclusions

In summary, we demonstrated that thoracic blast exposure can lead to CD28-related inflammation with increased levels of inflammatory factors, which leads to brain injury. Our data indicate that ROS generation, apoptosis, and activation of the PI3K/Nrf2/Keap1 signaling pathway play essential roles in the thoracic blast exposure-induced brain injury. Given the role of CD28 deficiency in protection against the above injuries, we suggest that blocking CD28-related signaling pathways will have therapeutic potential for the management of thoracic blast exposure-associated brain injury complications.

## Figures and Tables

**Table 1:** Primary antibody list.

	Dilution ratio	Catalogue number	Company
CD28	1 : 1000	#38774	Cell Signaling Technology
TRX	1 : 1000	ab86255	Abcam
MDA	1 : 1000	ab69983	Abcam
SOD-1	1 : 1000	ab16831	Abcam
SOD-2	1 : 1000	ab16956	Abcam
Bax	1 : 1000	ab32503	Abcam
Caspase-3	1 : 1000	ab32042	Abcam
Cytochrome C	1 : 1000	ab110325	Abcam
Bad	1 : 1000	ab32445	Abcam
Bcl-XL	1 : 2000	ab32370	Abcam
Nrf2	1 : 1000	ab137550	Abcam
Keap1	1 : 1000	ab66620	Abcam
Phospho-PI3 kinase p85 (Tyr458)	1 : 1000	#4228	Cell Signaling Technology
GAPDH	1 : 5000	#2118	Cell Signaling Technology

**Table 2:** Secondary antibody list.

	Dilution ratio	Catalogue number	Company
Anti-mouse secondary antibody	1 : 4000	ab6789	Abcam
Anti-rabbit secondary antibody	1 : 4000	ab6721	Abcam

## Data Availability

All the data used to support the findings of this study are included within the article.
